# Ferroptosis: A mixed blessing for infectious diseases

**DOI:** 10.3389/fphar.2022.992734

**Published:** 2022-09-07

**Authors:** Leyao Xiao, Huanshao Huang, Shuhao Fan, Biying Zheng, Jianguo Wu, Junai Zhang, Jiang Pi, Jun-Fa Xu

**Affiliations:** ^1^ Guangdong Provincial Key Laboratory of Medical Molecular Diagnostics, The First Dongguan Affiliated Hospital, Guangdong Medical University, Dongguan, China; ^2^ Institute of Laboratory Medicine, School of Medical Technology, Guangdong Medical University, Dongguan, China

**Keywords:** ferroptosis, mechanism, host immunity, infectious diseases, therapy strategy

## Abstract

To date, it has been confirmed that the occurrence and development of infectious diseases are tightly associated with regulatory cell death processes, such as apoptosis, autophagy, and necroptosis. Ferroptosis, as a newly discovered form of regulatory cell death characterized by iron-dependent lipid peroxidation, is not only closely associated with tumor progression, but is also found to be tightly related to the regulation of infectious diseases, such as Tuberculosis, Cryptococcal meningitis, Malaria and COVID-2019. The emerging critical roles of ferroptosis that has been found in infectious disease highlight ferroptosis as a potential therapeutic target in this field, which is therefore widely expected to be developed into new therapy strategy against infectious diseases. Here, we summarized the underlying mechanisms of ferroptosis and highlighted the intersections between host immunity and ferroptosis. Moreover, we illuminated the roles of ferroptosis in the occurrence and progression of different infectious diseases, which might provide some unique inspiration and thought-provoking perspectives for the future research of these infectious diseases, especially for the development of ferroptosis-based therapy strategy against infectious diseases.

## Introduction

Cell is the basic unit of the biological system, which not only provides the necessary energy and nutrition for physiological events, but also acts as the indispensable one for the host’s immunity against invasion. Various regulated cell death modalities are tightly associated with the pathological processes in many diseases. Strikingly, ferroptosis, a newly discovered cell death, accompanied by iron independence lipid peroxidation (LPO), has become an eye-catching topic nowadays (Dixon et al., 2012). The mechanism, morphology, and genomics of ferroptosis have been proved to be different from the well-known programmed cell death such as apoptosis, autophagy, and scorching death. Although lots of efforts have been made to depict the functions and mechanisms of ferroptosis, some critical mechanisms and related regulatory functions have not yet been fully explored, which requires more in-depth researches.

Over the past few years, shreds of evidences have shown that the close relationship between ferroptosis and cancer, neurodegenerative diseases, ischemia-reperfusion diseases, and kidney diseases (Stockwell et al., 2017; Stockwell et al., 2020; Wang et al., 2021b), however, there are relatively few studies describing the roles of ferroptosis in infectious diseases. Meanwhile, viruses, bacteria, and other pathogens in nature have coexisted with human beings for a long time, such as the world pandemic of COVID-19 (Kalra and Chawla, 2020), the growing bacterial drug resistance (Chen and Chen, 2021) and dangerous virus-related cancer (Ding et al., 2021) have brought endless suffering to people. Though many achievements and progresses have been made by human beings in the combat with infectious diseases, more works are still needed to be done, such as the updating of preventive measures and treatment strategies. Therefore, it is necessary to explore more in-depth understanding of the occurrence and development of infectious diseases. Potentially, ferroptosis may be a novel therapeutic target to develop more effective adjuvant therapies against infectious diseases.

It is conceivable, but not fully demonstrated, that ferroptosis triggered in infectious diseases acting a double-edged sword role that it may be caused by pathogens for survival or can be exploited as potential therapeutics. In this review, we briefly described the present understanding of ferroptosis induction and execution, and also highlighted the relationships between ferroptosis and infectious diseases so as to further understand the functions of ferroptosis in infectious diseases. Particularly, we hypothesized the therapeutic potential of ferroptosis in these diseases based on the current researches. We hope this review could enhance our understanding of ferroptosis in infectious diseases by exploring how ferroptosis contributes to host control of pathogens, how ferroptosis is triggered by some pathogens to promote disease development, and how ferroptosis can be controlled to defend against infectious diseases.

## Mechanism of ferroptosis

### Hallmarks of ferroptosis: Lipid peroxidation

**FIGURE 1:**
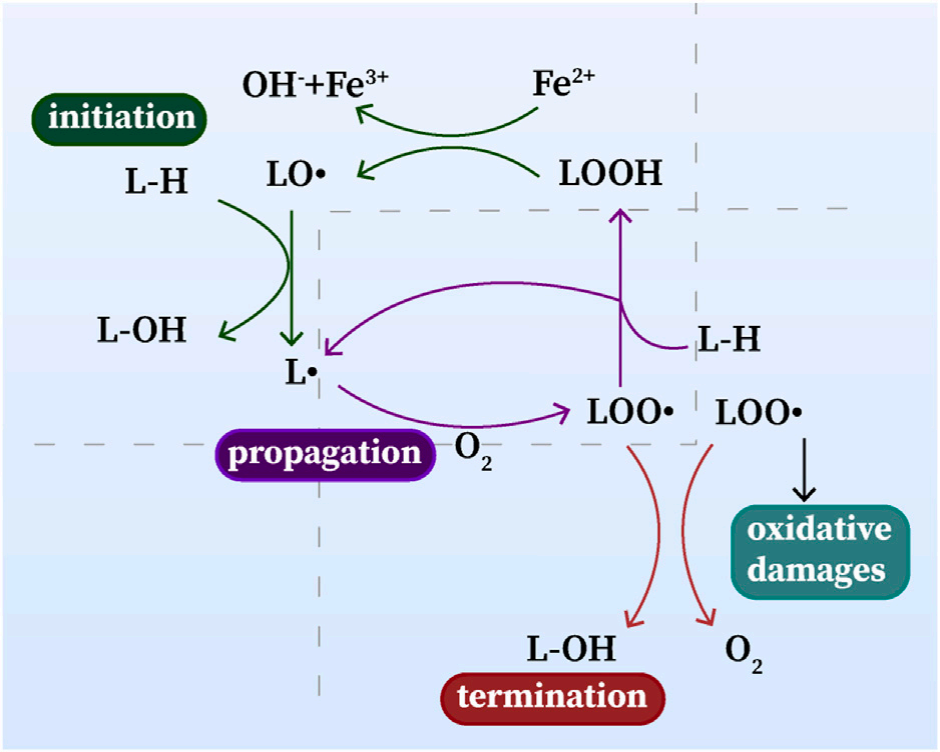
Lipid peroxidation: initiation, propagation and termination. L•: lipid radical; LO•: alkoxyl radical; L-OH, lipid alcohol; L–H, lipid; LOOH, lipid hydroperoxide; LOO•: epoxy peroxyl radical.

Lipid peroxidation is one of the most prominent features of ferroptosis, which is composed of a series of free radical chain reactions (Figure 1). Lipid peroxidation can be described generally as a process under which oxidants such as free radicals attack lipids containing carbon-carbon double bond(s), especially polyunsaturated fatty acids (PUFAs) (Minotti and Aust, 1989; Halliwell and Chirico, 1993; Cheng and Li, 2007). Free radicals, such as hydroxyl radicals (•OH) produced by the Fenton reaction (Fe^2+^ + H_2_O_2_ → Fe^3+^ + •OH + OH^−^), can be excessively produced due to an excess of ferrous iron (Cheng and Li, 2007). •OH is an essential substance for the initiation of lipid peroxidation and is capable of causing oxidative damage to cells. PUFAs are more susceptible to •OH compared with other intracellular lipids, the reaction between them generates LOOH, and ferrous iron catalyzes the cleavage of LOOH, again producing numerous ROS, such as LOO•, alkoxyl (LO•), or epoxy peroxyl radical (loo•), which further boost the oxidative stress in cell.(Halliwell and Chirico, 1993; Cheng and Li, 2007). As a result, this destructive lipid peroxidation disrupts the integrity and fluidity of the lipid bilayer of the cell membrane and ultimately leads to cell damage or death.

### GPX4 regulates ferroptosis

Glutathione peroxidase 4 (GPX4), a selenoprotein, one of the most well-known key factors in the regulation of ferroptosis, can reduce the level of intracellular lipid peroxide via lipids detoxification (Brigelius-Flohe and Maiorino, 2013; Yang et al., 2014). Glutathione (GSH) is used as a cofactor of GPX4 to assist GPX4, which converts toxic lipid hydroperoxides to non-toxic lipid alcohols. Thus, the reduction of GSH level will lead to the inhibition of GPX4, which suppresses the host capacity to repair peroxidized lipids and causes the occurrence of ferroptosis. Meantime, diverse ferroptosis inhibitors and inducers are applied to different kinds of research nowadays (Tables 1, 2) For example, as one of the representatives of class II ferroptosis-inducers (FINs) different from erastin, 1S, 3R-RSL 3(RSL3) can directly inhibit GPX4 without reducing the level of GSH to trigger ferroptosis. Knockdown of intracellular GPX4 can increase the level of intracellular lipid peroxides accompanied by ferroptosis, while iron chelator DFO and vitamin E prevent ferroptosis in GPX4 knockdown cells, reflecting the importance of GPX4 in protecting cells from excessive toxic lipid peroxides (Yang et al., 2014).

**TABLE 1:** Ferroptosis inducers.

Molecule	Target	Mechanism
Erastin Dixon et al. (2012)	System X_c_^-^	Block cystine import, cause GSH depletion
Sulfasalazine Dixon et al. (2014)	System X_c_^-^	Interfere cystine uptake, cause GSH depletion, lower potency than erastin
Glutamate Dixon et al. (2014)	System X_c_^-^	Higer extracellular concentrations prevent cystine import, causes GSH depletion
Sorafenib Dixon et al. (2014)	System X_c_^-^	Inhibit cystine import, cause GSH depletion
RSL-3 Yang et al. (2014)	GPX4	Bind to and inactivates GPX4
ML162 Moosmayer et al. (2021), ML210,JKE-1674 Eaton et al. (2020)	GPX4	Covalent inhibitor of GPX4
DPI7,10,12,13,17,18,19 Yang et al. (2014)	GPX4	Directly inactivate GPX4
FINO_2_ Gaschler et al. (2018)	GPX4	Indirectly inhibit GPX4 activity, oxidize labile iron
FIN56 Shimada et al. (2016)	GPX4	Degrade and inactivate GPX4
GPX4-IN-3(26a) Xu et al. (2021a)	GPX4	Inhibit GPX4, induce LPO
Dihydroisotanshinone I Wu et al. (2021a)	GPX4	Block GPX4 expression
Cisplatin Guo et al. (2018)	GSH	GSH depletion
Acetaminophen Lorincz et al. (2015)	GSH	GSH depletion
Ferric ammonium citrate Wu et al. (2021b)	GPX4-GSS/GSR-GGT axis	Induces oxidative injury
FAC (Fang et al., 2018)	Iron metabolism	Induces iron overload

**TABLE 2:** Ferroptosis inhibitors.

Molecule	Target	Mechanism
Deferoxamine, ciclopirox olamine Dixon et al. (2012)	Iron chelator	Iron chelation, suppress ROS accumulation
Thymosin β4 Lachowicz et al. (2022)	Iron chelator	Iron chelation, enhance anti-oxidative processes
Butylated hydroxytoluene, trolox Dixon et al. (2012)	LPO	Inhibit lipid peroxidation
GSK23344770 Fang et al. (2018)	LPO	Suppress RSL-induced lipid ROS production
Ferrostain-1 Skouta et al. (2014)	LPO	Inhibit the oxidative destruction of membrane lipid PUFAs, block lipid peroxidation
Lipoxstrain-1 Zilka et al. (2017)	LPO	Inhibit lipid peroxidation as RTAs
Nigratine Delehouze et al. (2022)	LPO	Inhibit phospholipid peroxidation, but a weak antioxidant compound
α-tocopherol, Vitamin E Hu et al. (2021)	LPO	Inhibit phospholipid peroxidation
N-Acetylcysteine Karuppagounder et al. (2018)	LPO	Neutralizes toxic lipids generated by ALOX5
Glutathione Yang et al. (2014)	LPO	Inhibit phospholipid peroxidation
β-ME Dixon et al. (2012)	Cystine uptake	Increases the cystine available for GSH synthesis, improves the activity of GPX4
Cycloheximide Dixon et al. (2012)	Protein synthesis	Suppress ferroptosis induced by system X_c_^−^ inhibitors
zileuton [32]	5-LOX	Inhibits 5-LOX

Due to the important roles of selenium in GPX4, there is also a close relationship between selenium and ferroptosis (Ingold et al., 2018). Selenoprotein GPX4 is composed of selenocysteine, which is similar in structure to ordinary cysteine, except that the sulfur is replaced by selenium at the active site of cysteine. Via replacing the active site of GPX4 selenocysteine with sulfur to cysteine, Ingold et al. found that the oxidative stress in the cell was intensified after adding H_2_O_2_, and the active site was oxidized to sulfonic acid (SO_2/3_H), which inactivated GPX4 and eventually induced ferroptosis. This work strongly suggests that selenium plays an indispensable role in helping GPX4 resist ferroptosis by regulating lipid peroxidation.

Moreover, Zhang Y et al. discovered a new pathway regulating GPX4 independently with the level of intracellular GSH (Zhang et al., 2021). Mammalian rapamycin complex 1 (mTORC1) plays an important role in regulating protein synthesis, cell growth, lipid metabolism, autophagy, and other biological activities. In the absence of intracellular cystine, the activity of mTORC1 will be inhibited and the amount of mTORC1 localized to lysosomes will be decreased, thereby reducing the level of intracellular GPX4.

Meanwhile, GPX4 is not the only intracellular antioxidant molecule inhibiting ferroptosis. Ferroptosis inhibitor protein 1 (FSP1) is an *in vitro* NADPH-dependent coenzyme Q10 (CoQ) oxidoreductase localized on the cell membrane and is able to inhibit lipid peroxidation in a different way (Bersuker et al., 2019). The supplementation of exogenous FSP1 could promote the reduced CoQ to enhance the antioxidant function of capturing free radicals, thereby inhibiting LPO and restoring the resistance of FSP1 knockdown cells to ferroptosis (Bersuker et al., 2019). Similar to GPX4 and FSP1, the lipid detoxification phospholipase iPLA2β can inhibit p53-induced ferroptosis by resisting lipid peroxidation (Chen et al., 2021), and avoid ferroptosis by reducing its substrate 15-HpETE-PE, a ferroptosis-promoting factor (Sun et al., 2021).

### Glutathione metabolism

Glutamine metabolism plays essential roles in cell biosynthesis, cell proliferation and cell death. The heterodimer of amino acid transporter solute carrier family 7 member 11 (SLC7A11) and amino acid transporter solute carrier family 3 member 2 (SLC3A2) is the main component of the sodium-dependent cystine/glutamate transporter (systemX_c_^−^) localized on cell membrane (Koppula et al., 2021), which exchanges cystine and glutamic acid at a ratio of 1:1. Cystine enters the cell and converts to cysteine for the synthesis of GSH, and SLC7A11 can regulate cystine uptake and participate in glutamine cycle metabolism (Zhang et al., 2021), thus promoting GPX4 protein synthesis. Consistently, Gao et al. illustrated that two specific amino acids, cystine and cysteine, were indispensable for the synthesis of intracellular GSH, and ROS was increased once in the absence of them (Gao et al., 2015). Meantime, glutamate-cysteine ligase catalytic subunit (GCLC), a catalytic enzyme involved in GSH synthesis pathway, can repress the sensitivity of cells to cystine and cysteine deficiency-induced ferroptosis (Kang et al., 2021). In addition, BECN1 (beclin 1) can directly bind to SLC7A11 and inhibit its function for the uptake of cystine, which leads to the lack of intracellular GSH and the accumulation of lipid peroxidation, triggering ferroptosis eventually (Song et al., 2018).

#### Iron metabolism

Iron is required for the execution of ferroptosis, thus, iron homeostasis is inseparable from the occurrence of ferroptosis. To date, a variety of related molecules have been found to regulate and maintain intracellular iron homeostasis (Halliwell and Chirico, 1993; Anderson and Vulpe, 2009). Transferrin receptor (TfR) is a prominent molecule for storing excess intracellular iron. The deficiency of TfR is capable of stimulating chronic iron accumulation and increasing the sensitivity of cells to ferroptosis. For example, the lack of TfR in hepatocytes is accompanied by ferroptosis, which aggravates the degree of cell damage (Yu et al., 2020).

Poly(C)-binding proteins 1 (PCBP1), an iron chaperone from one of the four homologous RNA-binding protein families in KH domain superfamily, can directly bind iron and combine with ferritin to complete the storage of intracellular iron (Shi et al., 2008). Evidence showed that the lipid peroxidation and product 4-Hydroxynonenol (4-HNE) were increased in PCBP1 knockout mouse liver cells and the level of GPX4 was also increased accordingly (Protchenko et al., 2021). Knockout of PCBP1 led to the failure of its function as an iron chaperone, and the increased free ferrous iron in the cell thus catalyzed the production of ROS through the Fenton reaction, resulting in the accumulation of lipid peroxides. This research also pointed out that PCBP1 could control the redox response in labile iron pool (LIP), thereby inhibiting iron-induced LPO or cell death under physiological conditions. Importantly, PCBP1 has the ability to repress ferritinophagy-mediated ferroptosis via inhibiting ferritinophagy through silencing BECN1 mRNA and binding with ALOX15 to attenuate the susceptibility of cells to ferroptosis (Lee et al., 2022).

In recent years, the relationship between autophagy and ferroptosis has engaged much attention, and the view that ferroptosis is an autophagy-dependent death is emerged. Many studies have reported that ferritinophagy can trigger ferroptosis (Hou et al., 2016; Zhou B. et al., 2020). Ferritin, an important protein for storing iron (Theil, 2004), can avoid cell damage caused by Fenton reaction via combining with excess ferrous iron (Minotti and Aust, 1989; Halliwell and Chirico, 1993; Cheng and Li, 2007). Notably, nuclear receptor coactivator (NCOA4), the selective carrier receptor of ferritinophagy, can drive ferritinophagy via releasing excess iron into the cell to induce autophagy degradation of ferritin (Mancias et al., 2014). Physiologically, the intracellular iron content can be compensated by ferritinophagy via the NCOA4 pathway. At the same time, down-regulation of NCOA4 prevents excessive ROS production to inhibit the occurrence of ferritinophagy and ferroptosis, while over-expression of NCOA4 causes the opposite performance. What’s more, the hypoxic environment can inhibit NCOA4-regulated ferroptosis and avoid cell death (Fuhrmann et al., 2020).

Meanwhile, a paper proposed that ATG5, an autophagy-related gene, regulated ferritinophagy and then induced ferroptosis (Hou et al., 2016). Eunhee Park et al. subsequently confirmed this view, they discovered that erastin could induce autophagy-related cell death, which further lead to iron-dependent ferroptosis by degradation of ferritin and induction of TfR1 (Park and Chung, 2019). Also, the inhibition of autophagy can downregulate intracellular iron content and weaken the degree of lipid peroxidation in ferroptosis, illuminating that ferritinophagy is a vital process involved in ferroptosis. Meanwhile, they considered the occurrence of autophagy as the consequence for the increase of intracellular ROS induced by erastin. Moreover, another group (Ma et al., 2017) indicated that the increase of iron-dependent ROS could also cause a similar phenomenon, suggesting that the inducement of autophagy in ferroptosis can be diversified. In addition, ferroptosis and autophagy can also occur at different time periods, implying that ferroptosis can also occur independently from autophagic cell death.

#### Lipid metabolism

Acyl-CoA synthetase long-chain family member 4 (ACSL4) has been confirmed as a key ferroptosis gene, playing a crucial role in the synthesis of long-chain PUFA-CoA (Doll et al., 2017; Kagan et al., 2017). Pharmacological inhibition of ACSL4 in tumor cells has been proved to show inhibition effects on ferroptosis. Also, knocking out ASCL4 in cells reduced the level of PUFAs and eliminated the inhibitory effect of RSL3 on GPX4, thereby inhibiting ferroptosis (Doll et al., 2017), implicating that ASCL4 played a key role in RSL-3-induced ferroptosis. Notably, ASCL4 has the ability to enrich cell membrane lipids, thereby increasing the susceptibility in cell to ferroptosis. Moreover, research showed that protein kinase PKCβII, a lipid peroxide sensing molecule, promoted and amplified ferroptosis-related lipid peroxidation by phosphorylating ASCL4 and accelerated the occurrence of ferroptosis (Zhang H. L. et al., 2022). Meanwhile, Cytochrome P450 oxidoreductase (POR) can also catalyze lipid peroxidation of PUFAs, thereby promoting ferroptosis (Zou et al., 2020).

Lipoxygenase (LOX) catalyzes the production of lipid peroxides, as proof, Arachidonate 12-Lipoxygenase (ALOX12), an isoform of the mammalian lipoxygenase family, can inhibit p53-regulated ferroptosis via inhibiting lipid synthesis function by specifically binding to SLC7A11, which abolishes the function of p53 suppressing tumor growth through ferroptosis (Chu et al., 2019). Also, over-expression of 5-LOX, p12-LOX, and 15-LOX-1 could enhance cellular susceptibility to ferroptosis by catalyzing lipid peroxidation, but these LOXs are likely to boost the initial stage of ferroptosis by promoting the formation of lipid hydroperoxides (Shah et al., 2018).

#### Energy stress

What’s more, cellular energy stress might also be closely bound up with ferroptosis. For instance, activation of AMP-activated protein kinase (AMPK) caused by energy stress can inhibit the synthesis of certain anabolism such as PUFAs, which further suppresses ferroptosis (Lee et al., 2020). However, another study showed opposite results, which pointed out that AMPK helped BECN1 inhibit the transport of cystine by SCL7A11, and ultimately provoked ferroptosis (Song et al., 2018). These two opposite findings and the differences in the mechanisms involved may not be sufficient to clarify the precise role of energy stress in ferroptosis, but both indicated the critical roles of energy stress in ferroptosis.

#### p53-mediated ferroptosis

Intriguingly, p53 was firstly found to suppress tumor growth via the induction of ferroptosis instead of the canonical way like apoptosis (Jiang et al., 2015). They illustrated that p53 inhibited cystine uptake by repressing the expression of SLC7A11, which sensitized cells to ferroptosis. Significantly, Jiang et al. not only proposed a new mechanism of tumor suppression but also disclosed the latent relationship between p53 and ferroptosis. More importantly, this non-canonical p53 activities provides a brand-new perspective for other researches in many diseases. Also, they further uncover that p53 acetylation is crucial for p53-Mediated ferroptosis and tumor suppression (Wang et al., 2016). However, it is still a mystery that how p53 orchestrates the ferroptotic responses while it executes its mission. Later, it was confirmed that SAT1 (spermidine/spermine N^1^ -acetyltransferase 1) gene induced by p53 could trigger lipid peroxidation and sensitize cells to ferroptosis (Ou et al., 2016). Moreover, ALOX15 induced by SAT1 is the executioner of the occurrence of ferroptosis. Meanwhile, p53-ALOX12 axis as we mentioned before and BRD7 (bromodomain-containing protein 7)-P53-SLC25A28 (solute carrier family 25 member 28) axis is also a part of the regulation mechanism of p53-induced ferroptosis (Chu et al., 2019; Zhang et al., 2020).

However, p53 can play a double-edged sword role in mediating ferroptosis. p53 stabilization suppress ferroptosis in response to systemX_c_^−^ inhibition, which is different from previously identified function of p53 as a positive regulator of ferroptosis (Tarangelo et al., 2018). At the same time, TP53 surprisingly limits erastin-induced ferroptosis via blocking dipeptidyl-peptidase-4 (DPP4) activity (Xie et al., 2017). Increasing studies about p53-related ferroptosis have been published (Liu and Gu, 2022), gradually becoming an indispensable puzzle piece of the whole quest to explore the mechanisms of ferroptosis.

**FIGURE 2:**
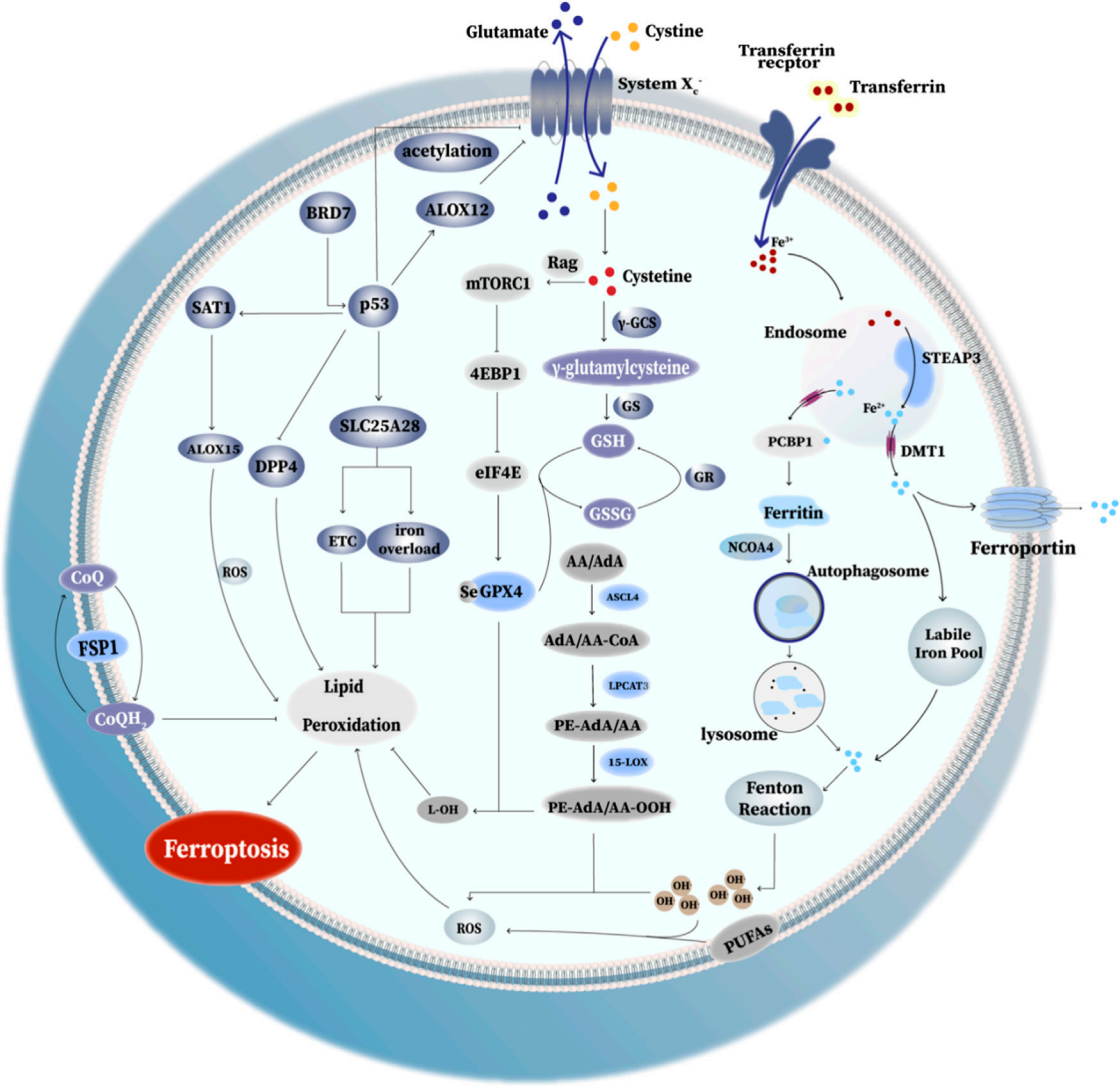
Mechanism of ferroptosis. I. Ferritinophagy-related ferroptosis: the degradation of ferritin via autophagy causes the iron dysregulation, which can lead to iron overload and trigger ferroptosis. II. GPX4 anti-ferroptosis way: GPX4 converts toxic lipid hydroperoxides to non-toxic lipid alcohols. SystemX_c_^−^ exchanges cystine and glutamate in a ratio of 1:1. Cystine enters the cell and converts to cysteine for the synthesis of GSH, thus promoting GPX4 protein synthesis and enhancing its antioxidant function. III. Rag-mTORC1-4EBP signaling axis: mammalian target of rapamycin complex 1 (mTORC1) and promotes GPX4 protein synthesis at least partly through the Rag-mTORC1-4EBP signaling axis. (Rag(Ras-related GTPase): Rag GTPases play important roles in mTORC1 activation in response to amino-acid stimulation; eIF4E: eukaryotic initiate factor 4E; 4EBP(eIF4E binding protein): binding to eIF4E and thus decreased GPX4 level. IV. p53-mediated ferroptosis: (1) acetylation of p53 is crucial for p53-induced ferroptosis; (2) SAT1 activated by p53 induces ferroptosis via promoting ALOX15 expression;(3) the elevated BRD7 expression promote p53 mitochondrial translocation, leading to the interaction between mitochondrial p53 and SLC25A28, which could lead to the abnormal accumulation of redox-active iron and hyperfunction of electron transfer chain (ETC). (4) p53 promote ALOX12 binding with SLC7A11, eventually causing the inhibition of SystemX_c_^−^ and triggering ferroptosis. (5) p53 inhibit erastin-induced ferroptosis via blocking the activity of DPP4. V. FSP1: FSP1 locates in the plasma membrane where it functions as an oxidoreductase that reduces coenzyme Q_10_ (CoQ). Reduced CoQ acts as a lipophilic radical-trapping antioxidant that halts the propagation of lipid peroxides, thus inhibiting ferroptosis. VI. PE-OOH as a ferroptotic death signal: ferroptosis involves a highly organized oxygenation center, wherein oxidation in endoplasmic-reticulum-associated compartments occurs on only one class of phospholipids (phosphatidyletha- nolamines (PEs)) and is specific toward two fatty acyls—arachidonoyl (AA) and adrenoyl (AdA). Moreover, several key enzymes like ASCL4, LPCAT3 and 15-LOX play a key role in proferroptotic system.

To date, mounting evidence has showed that the mechanisms and functions of ferroptosis (Figure 2) and its complicated and diverse relations with mutiple diseases. Intriguingly, these mechanisms may shed light on therapeutic development in different diseases in the future.

### Ferroptosis and phagocytosis

#### Ferroptosis and DAMPs

Innate immune cells, the first line of defense against infection, recognize pathogen-related molecular patterns (PAMPs) through pattern recognition receptors (PRRs) (i.e., Toll-like receptors (TLRs) and NOD-like receptors (NLRs)), thus initiating basic, simple, and rapid defensive responses to fight pathogen invasions (Thaiss et al., 2016). Damage-associated molecular patterns (DAMPs) (i.e., high mobility group protein B1 (HMGB1)) can be released from the host to initiate the corresponding inflammatory response against infections (Zindel and Kubes, 2020), such as regulating the inflammatory responses induced by ferroptosis (Wen et al., 2019). HMGB1 could increase through autophagy and bind to advanced glycosylation end-product specific receptor (AGER) to promote macrophage release tumor necrosis factor (TNF) in response to erastin, sorafenib, RSL3, and FIN56 (ferroptosis inducer 56) induced ferroptosis. In addition, knockdown of HMGB1 reduced erastin-induced ROS generation and suppressed TfR1 expression through the RAS-JNK/p38 pathway, thereby regulating ferroptosis (Ye et al., 2019).

#### Ferroptosis and macrophages

TLRs are able to recognize PAMPs and DAMPs, and initiate corresponding immune responses, such as the immune clearance function of macrophages. Research believed that the efficiency of macrophages to clear apoptotic cells was better than that of ferroptotic cells (Kloditz and Fadeel, 2019). However, there is also research considered that SAPE-OOH (1-steaoryl-2-15-HpETE-sn-glycero-3-phosphatidyletha- nolamine), an oxidized phospholipid molecule on the membrane of ferroptotic cells, could be specifically recognized by TLR2, and thus provoked macrophages to phagocytose ferroptotic cells as immune clearance (Luo et al., 2021). Meanwhile, they found that macrophages in TLR2 knockout mice also phagocytosed ferroptotic cells well, suggesting that in addition to TLR2 recognition of SAPE-OOH, there were other pathways to maintain the phagocytic clearance of ferroptotic cells. Significantly, Gao’s team observed that ferroptosis inducers could induce ferroptosis in intracellular bacteria, thereby assisting macrophages to inhibit the growth of intracellular bacteria (Ma et al., 2022). In addition, they also dynamically monitored ferroptosis markers in macrophages after 12 and 24 h of bacteria infection and discovered that these markers increased during early infection, but dropped back to normal at the late stage. These results imply that ferroptosis has the potential to be served as a potential therapeutic target for intracellular bacteria at the early stage of infection.

What’s more, with the stimulation of pathogen infection, TLR recognition, and interferon signal regulation, macrophages usually differentiate into M1 macrophages equipped with pro-inflammatory effects, while M2 macrophages have the anti-inflammation and tissue repair effects (Murray, 2017). Also, a study showed that KRAS protein-encapsulated exosomes, secreted by pancreatic ductal adenocarcinoma mouse cancer cells that mediated by autophagy-dependent ferroptosis, could combine with AGER on the surface of macrophages, eventually promoting the differentiation of macrophages toward M2 (Dai et al., 2020). While macrophages perform functions such as endocytosis, the cells in aerobic environment yet can’t lead to ferroptosis in M1 macrophages, because there are a large number of inducible nitric oxide synthase (iNOS) in the activated M1 cells, which plays an antioxidant role similar to GPX4 (Kapralov et al., 2020). The high level of iNOS eventually makes M1 macrophages be resistant to ferroptosis, while conversely, M2 macrophages who lack iNOS is more sensitive to ferroptosis. At the same time, M1 macrophages can exert this protective function to protect neighboring cells from ferroptosis by regulating their resistance. Ferroptosis-related metabolism, such as glutathione metabolism, also has an impact on macrophage polarization. Adequate glutamine contributes to M2 differentiation, if glutamine is deficient, the number of M2 cells would decrease with the down-regulation of related genes, leading to the significant down-regulation of TCA (tricarboxylic acid) cycle transcriptional activity and autophagy disorders (Jha et al., 2015). Obviously, the relationship between ferroptosis, ferroptosis-related metabolism and macrophage polarization, and the ability to resist infection require more detailed researches.

One might wonder whether there are different correlations between macrophage and ferroptosis. Indeed, macrophages not only play a momentous role in anti-infection immunity but also gobble up aging or injured red blood cells and regulate iron homeostasis. Different polarization types of macrophages come with different functions in regulating iron metabolism (Cairo et al., 2011). It is widely known that iron contributes to the growth of invading bacteria. Under the stage of infection, M1 macrophages actively save iron in cells to limit the growth of extracellular bacteria by reducing the expression of ferroportin1 (FPN1), the only known iron exporter, and increasing extracellular iron uptake (Ganz, 2009). Consistently, iron deficiency can inhibit the growth of intracellular bacteria (Paradkar et al., 2008). For example, IFN-γ (Interferon-γ) limits the number of intracellular bacteria by reducing iron intake and increasing FPN1 expression (Nairz et al., 2008). In addition, contrary to the function of M1 macrophages, M2 macrophages tend to release iron to extracellular cells through FPN1 (Recalcati and Cairo, 2021).

#### Ferroptosis and neutrophil

Neutrophil is another important member of innate immune system, which counteracts pathogens via some mechanisms, such as phagocytosis and the production of NETs (neutrophil extracellular traps) (Castanheira and Kubes, 2019). Many published papers have told us the correlations between ferroptosis and neutrophil in cancer, but there are still limited information in infectious diseases. Proverbially, NETs are weapons used by neutrophils to fight against microbes (Brinkmann et al., 2004). However, in sepsis-associated acute lung injury (ALI), NETs contribute to the pathological progression through inducing ferroptosis in alveolar epithelial cells (Zhang H. et al., 2022).

A paper illustrating precise cell death pathways and signaling events orchestrate early inflammation after heart transplantation is thought-provoking (Li et al., 2019). They suggested that ferroptosis initiated neutrophil recruitment through TLR4/Trif signaling pathways, which reflected the possibilities of ferroptosis and relevant signaling events affecting innate immune function. Also, neutrophil-triggered ferroptosis occurred in tumor cell and showed positive feedback for tumor progression (Yee et al., 2020). These works collectively suggested that there might also be some critical roles for neutrophil-triggered ferroptosis against infections, which still need to be further confirmed.

### Ferroptosis and infectious diseases

#### Ferroptosis and viral infection

**FIGURE 3:**
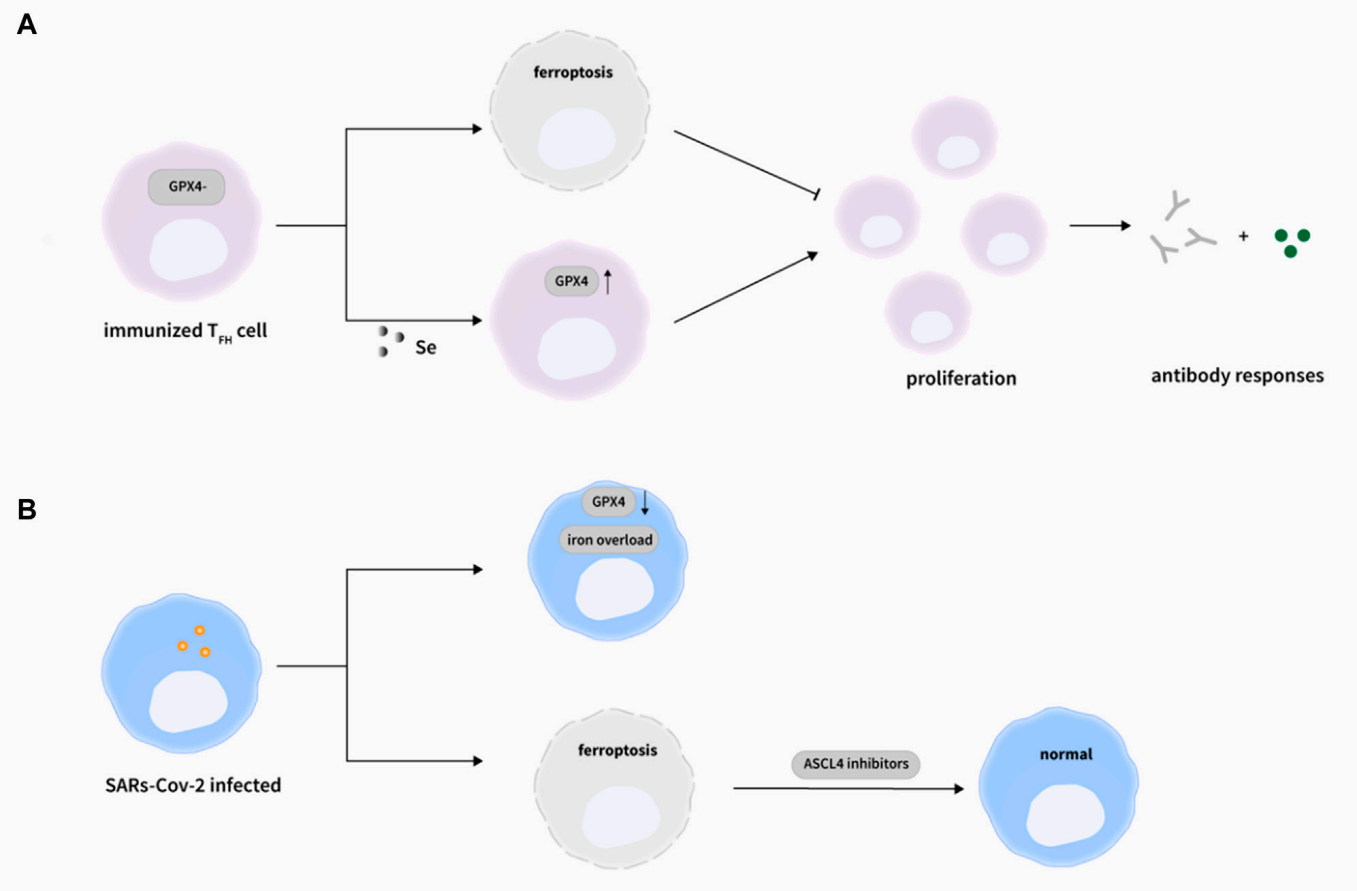
Ferroptosis and viral infections. **(A)** The deletion of GPX4 in T cells selectively abrogated T_FH_ cells functions via ferroptosis in immunized mice. Importantly, selenium supplementation cloud enhance GPX4 expression in T cells, promoting T_FH_ cell proliferation and boosting antibody responses in immunized mice. (Yao Y. et al., 2021). **(B)** During SARs-Cov-2 infection, the Gpx4 mRNA level was decreased (Wang et al., 2021a) and iron overload was observed (Zhou C. et al., 2020), and the induction of ferroptosis by SARS-Cov-2 could be rescued by ASCL4 inhibitors (Kung et al., 2022)

Tremendous progress has been made to defend against pathogens invasion for a long time, a variety of vaccines have been developed to guard against virus infection or to reduce the symptoms of infection, but not all individuals can produce high titers of antibodies to successfully prevent disease after vaccination. Proverbially, T cell immunity is indispensable for defending against virus invasion. Matsushita et al. found that the immune function and virus clearance ability of GPX4-deficient T-cell mice were impaired after being infected with lymphocytic chorititis virus (Matsushita et al., 2015). Here, GPX4 deficiency caused abnormal lipid peroxidation, which led to T cell ferroptosis, thus weakening T cell immunological responses against virus infection. However, vitamin E, which has an antioxidant function, could restore the number of T cells in mouse and enhance their antiviral response. After that, Yao’s team reported that the occurrence of ferroptosis in follicular helper T(T_FH_) cells in mouse and human tonsils after ovalbumin injection, with higher levels of lipid ROS, MDA (malondialdehyde), and 4-HNE in T_FH_ cell compared with non-T_FH_ cells (Yao Y. et al., 2021). In addition, the same phenomenon was also found in human peripheral blood. Furthermore, combined with the result of Matsushita et al. (Matsushita et al., 2015), they listed some opinions: I. GPX4 selectively takes part in T_FH_ cell immune responses, II. Enhanced TCR signal increases the sensitivity of T_FH_ cell to ferroptosis, III. T_FH_ cell immune response can be regulated by ferroptosis. Also, with the treatment of selenium, the GPX4 expression and the number of T_FH_ cell were increased, and the efficiency of antibody responses in mice and teenagers after vaccination were elevated. In summary, these results reveal that “selenium-GPX4-ferroptosis” plays a crucial role in regulating T_FH_ cell homeostasis, and can be targeted to enhance the immune response of the human body after vaccination, providing ideas and possibilities for preventing viral infection and enhancing the immune effects of the vaccine (Figure 3A). What’s more, antigen-specific memory CD4^+^ T cells can persist and confer rapid and efficient protection from microbial reinfection. mTORC2 (Mammalian rapamycin complex 1) is critical for long-term persistence of virus-specific memory CD4^+^ T cells, which ablation will induce aberrant mitochondrial ROS accumulation and ensue ferroptosis-causative lipid peroxidation (Wang et al., 2022).

Meanwhile, the expression of p53 in U251 cells infected by Newcastle-disease-virus (NDV) was up-regulated, which led to the decrease of SLC7A11 and GPX4 protein levels, while knockdown of p53 reduced the ROS level (Kan et al., 2021). And NCOA4-related ferritinophagy was triggered by NDV, which could increase the level of intracellular ROS and ferrous iron, and then trigger ferroptosis, indicating that NDV could kill tumor cells in a ferroptotic way by inducing ferritinophagy or inhibiting SLC7A11.

What’s more, lipid metabolism is involved in viral replication by regulation of the formation, assembly, and release of replicative organelles. ASCL4, a key factor involved in lipid metabolism and ferroptosis, greatly promoted the replication of some enteroviruses and coronaviruses (Kung et al., 2022). In ASCL4 knockout cells, the viral titers of enteroviruses such as CV-A6, coronaviruses such as Cov-229E, influenza virus (IAV), and Zika virus were lower than those in the normal cells. In addition, enterovirus CV-A6 and coronaviruses such as CoV-229E, CoV-NL63, CoV-OC43, CoV-HKU1, and SARS-CoV-2 could induce ferroptosis in cells by regulating ASCL4 functions. Consistently, the inhibition of ASCL4 can repress virus replication effectively by inhibiting ferroptosis, which implicates the significance of ASCL4-induced ferroptosis in viral replication.

Previous studies have implied that ferroptosis might participate in the pathogenesis of COVID-19 (Figure 3B), as proof, iron overload was relatively associated with the COVID-19 infection (Zhou C. et al., 2020). Increased hepcidin may raise the risk of ferroptosis occurrence by iron accumulation in cellular, which certainly suggest the therapeutic potential of iron chelators. Ferroptosis inhibitors includes iron chelators, like the famous one DFO (deferoxamine), has been approved by the FDA for the treatment of iron overload (Meyer, 2006). Meanwhile, the induction of ferroptosis by SARS-Cov-2 could be rescued by ASCL4 inhibitors (Kung et al., 2022). Also, SARS-Cov-2 showed inhibitory effects on the expression of the selenoprotein GPX4 mRNA level *in vivo*(Wang et al., 2021a). Undoubtedly, targeting ferroptosis will pose some new perspectives for preventing and controlling viral infections. At the same time, the influence of SARS-CoV-2 and possible immunosuppressive drugs can cause the temporal inhibition of the humanbody immunological function, thus resulting in active TB caused by reactivation or infection of *M. tuberculosis* (Yang and Lu, 2020). Consequently, the co-infection of different pathogens requires more attentions in the future.

#### Ferroptosis and bacterial infection

**FIGURE 4:**
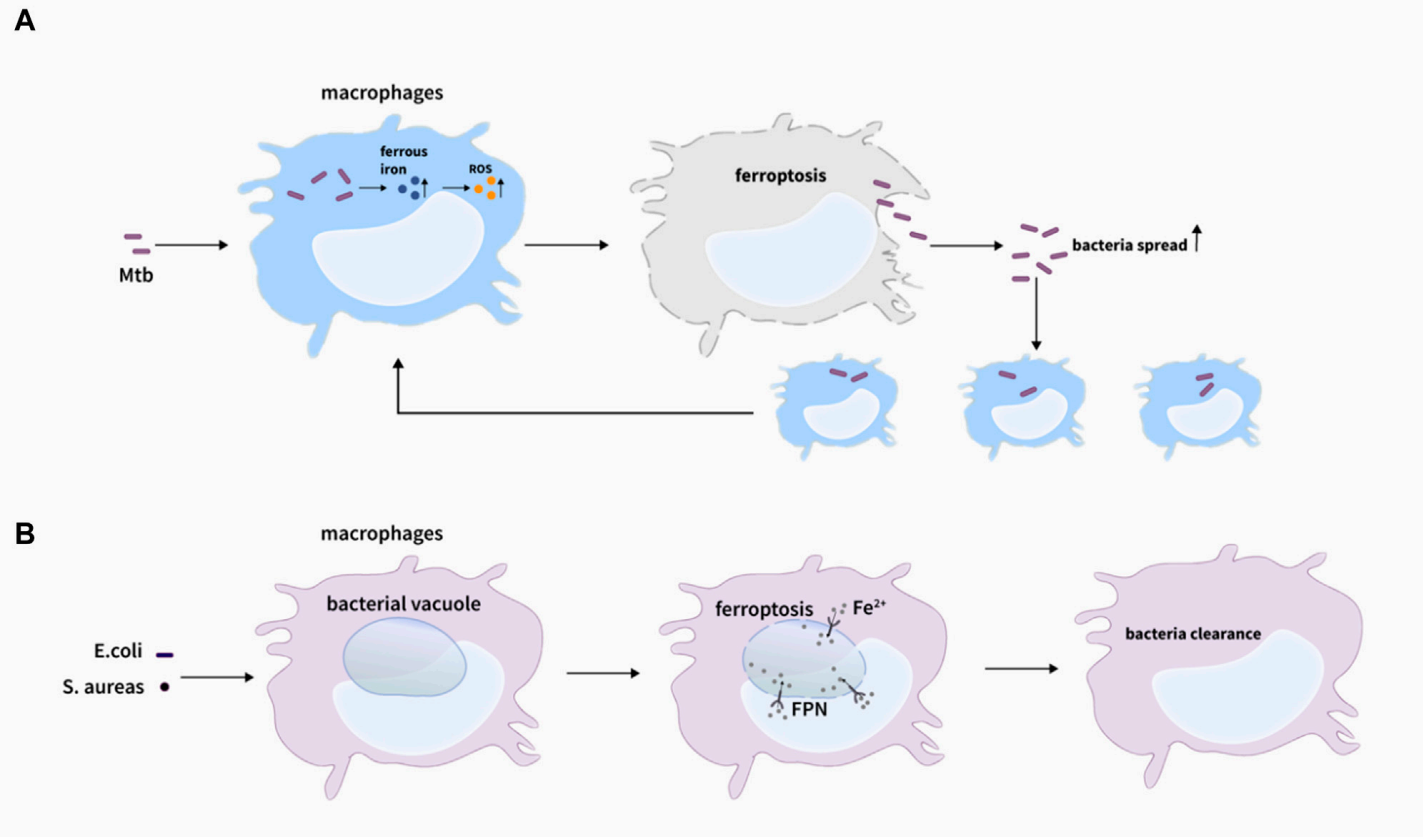
Ferroptosis and bacteria infections. **(A)** Lipid peroxidation induces plasma membrane destabilization, leading to ferroptosis-mediated cell death with *M. tuberculosis* infection, and ferroptosis drives macrophages necrosis and allows *M. tuberculosis* to thrive and spread, which promote the infection (Amaral et al., 2019). **(B)** Ferrous iron could be delivered to the intracellular bacterial vacuole via inward FPN transportation, eventually inducing ferroptosis-like death of bacteria, which assists killing bacteria of macrophages (Ma et al., 2022).

Previous studies have focused on the relationship between iron and *Mycobacterium tuberculosis* (Mtb), although excess iron could be extremely toxic to Mtb (Imlay et al., 1988; Byrd, 1997; Rodriguez, 2006), sufficient iron to some extent is beneficial for the growth and propagation of Mtb in the host (Gangaidzo et al., 2001; Schaible and Kaufmann, 2004). High concentrations of vitamin C could inhibit the growth of MDR (multiple drug resistance)-Mtb in the medium, leading to an increase of iron concentration in the bacteria with more ROS generated through the Fenton reaction, which in turn promoted lipid synthesis, caused DNA damage, changed the redox homeostasis, and ultimately inhibited the growth of MDR-Mtb (Vilcheze et al., 2013). However, Amaral et al. firstly discovered that Mtb-induced macrophage necrosis had many characteristics of ferroptosis, accompanied by iron overload, lipid peroxidation, and GPX4 downregulation in H37Rv-infected macrophages in both vivo and *in vitro*, which greatly increased the number of cell death (Amaral et al., 2019) (Figure 4A). And importantly, a reduction in granulomatous inflammation was observed in tissue sections with the treatment of ferrotstain-1 (Fer-1), and bacterial load in the lung of mice was also decreased, suggesting that inhibition of ferroptosis might contribute to the host cell resistance to Mtb to a certain extent. Meanwhile, BACH-1, a transcription factor, can disrupt iron homeostasis and redox by inhibiting GSH synthesis or the expression of LIP homeostasis-related genes, thereby promoting ferroptosis (Nishizawa et al., 2020). Combined with this, they observed that the number of intracellular bacteria and lung necrosis areas were reduced after the knockout of BACH-1 (Amaral et al., 2020). Consistently, the deficiency of BACH-1 in iron overload and Mtb-infected macrophages could resist the impairment of iron overload and ferroptosis (Aberman et al., 2021). Commonly, the above studies suggest that BACH-1-related ferroptosis may be a potential target for the effective treatment of tuberculosis. But interestingly, ferroptosis can also help macrophages kill intracellular bacteria such as s.aureas (Ma et al., 2022) (Figure 4B), which is contrary to the above studies to some degree, indicating that the mechanism of ferroptosis in macrophage resistance to intracellular bacteria still needs to be further explored.

Commonly, 15-lipoxygenases (15LOXes) can induce ferroptosis by catalyzing lipid peroxidation (Kagan et al., 2017). In general, lipoxygenase can’t be produced in bacteria, but surprisingly, 15LOXes can be expressed in *P. aeruginosa* (Vance et al., 2004). Moreover, Dar et al. firstly proposed that 15-LOXes in *P. aeruginosa* could catalyze lipid peroxidation in human bronchial epithelial cells, thereby inducing ferroptosis and further spreading to surrounding cells and tissues (Dar et al., 2018). Oxidative stress caused by *P. aeruginosa* is one of the most important causes of cystic fibrosis in the human lung as *P. aeruginosa* can elevate ROS in cystic fibrosis airway epithelial cells. This could lead to lipid peroxidation and ultimately trigger ferroptosis, while Fer-1 and other ferroptosis inhibitors could improve the pathological condition of the diseased airway (Ousingsawat et al., 2021), implicating that the inhibition of ferroptosis was of great significance in the treatment of *P. aeruginosa.*

In addition, some studies have also revealed the important role of ferroptosis in the development of sepsis. For example, the inhibition of lipopolysaccharide-induced ferritinophagy-related ferroptosis could improve cardiac function and survival prognosis in mice with lipopolysaccharide-induced cardiac injury (Li et al., 2020). At the same time, itaconate, a metabolite produced during inflammatory macrophage activation, could inhibit ferroptosis by up-regulating Nrf2 (Nuclear Factor erythroid 2-Related Factor 2) levels, thereby alleviating the symptoms of acute lung injury (ALI) and the presence of macrophages in the lung tissue infiltration (He et al., 2022).

#### Ferroptosis and fungal infection

Cryptococcal meningitis (CM) is one of the most common clinical fungal infections, especially in AIDS patients with immunodeficiency. Notably, iron accumulation and lipid peroxidation occurred in the brains of CM patients (Xu X. et al., 2021), and ferritin levels in the cerebrospinal fluid were significantly elevated (Campbell et al., 1986). Consistently, iron overload exacerbates the condition of CM patients (Barluzzi et al., 2002). Meantime, after Cryptococcus infection of alveolar macrophages, significant intracellular lipid peroxidation occurred, and a large number of high-density lipid droplets were observed under electron microscopy (Gross et al., 2000). In addition, Cryptococcus infection of activated macrophages in the cerebrospinal fluid of AIDS patients could promote the release of pro-inflammatory cytokines and chemokines, which further promoted the expression of DMT1 and FPN1, and indirectly increased the extracellular iron uptake level of cells, resulting in an intracellular iron surplus (Urrutia et al., 2013; Jarvis et al., 2015). To date, no studies have directly pointed out the role of iron excess and lipid peroxidation in cryptococcus infection. However, considering that excess iron can induce lipid peroxidation, it can be assumed that there may be potential connections among iron accumulation, ROS accumulation, ferroptosis, and cryptococcus infection, which requires further explorations.

**FIGURE 5:**
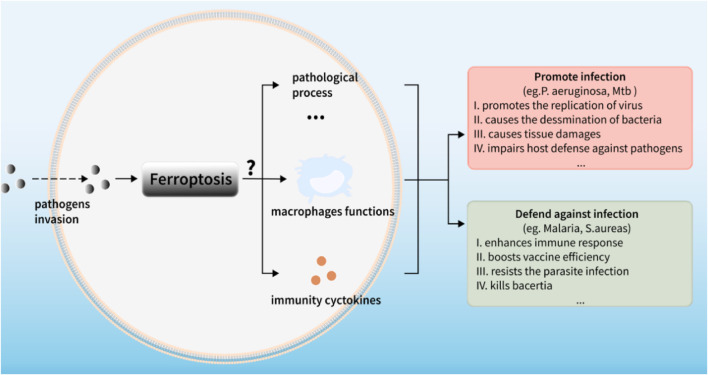
Ferroptosis: a mixed blessing for infectious diseases.

Iron is of great significance both to the invading of bacteria and fungi in human body. Patients with clinical liver transplantation are prone to fungal infection, which seriously threatens the patients. A retrospective study suggested that iron overload was an independent risk factor for fungal infection after liver transplantation, and controlling iron content before transplantation could reduce the risk of fungal infection (Alexander et al., 2006). Also, unlike ferroptosis regulated by Fenton response mediated by LOXs or Fe^2+^, NOXs can regulate ferroptosis induced by exogenous Fe^2+^ in Aspergillus flavus (Yao L. et al., 2021). However, there are few published researches that uncover the links between ferroptosis and fungal infections, such as Candida, Aspergillus, and Saccharomyces infections. Obviously, more works are needed to done to further explore this terra incognita.

#### Ferroptosis and parasitic infection

Infection of GPX4-deficient mice with Leishmania could lead to a reduction in the number of CD4^+^ T cells, which contributes to the maintenance of Leishmania *in vivo* (Matsushita et al., 2015). Malaria caused by Plasmodium infection in humans is prevalent all over the world, especially in Africa. Hence, the elimination of malaria is particularly important for the public health (Cotter et al., 2013). Once infected with malaria parasites, the host-owned pattern recognition receptors recognize the PAMPs and DAMPs of the parasite and activate immune cells to secrete inflammatory factors, causing oxidative stress to generate a large amount of ROS, which may eventually lead to cell death (Cotter et al., 2013). What’s more, Singh et al. reported that the mutation of amino acid 47 in human TP53 caused defects in p53-induced ferroptosis, resulting in massive intracellular iron accumulation in macrophages, which would lead to M2-polarization of macrophages, eventually helping resist Plasmodium infection but also promoting bacterial infections such as Listeria (Singh et al., 2020). In addition, malaria parasites can obtain sufficient iron from hemoglobin by attacking red blood cells, but can also lead to excess ferrous iron, exacerbating the Fenton reaction (Sena-Dos-Santos et al., 2021). Obviously, Plasmodium infection is closely related to iron homeostasis and ROS, and intracellular iron metabolism is important for resistance to Plasmodium. Notably, the SLC7A11-GPX4 pathway helps resist the infection of liver Plasmodium (Kain et al., 2020). And p53 indirectly suppresses the activity of GPX4 via inhibiting SLC7A11, causing lipid peroxidation to kill Plasmodium. Apparently, Plasmodium is sensitive to ferroptosis, and induction of ferroptosis can protect against Plasmodium infection to a certain extent.

### Therapeutic potential of ferroptosis

Nowadays, glucocorticoids are used as clinical routine to treat COVID-19, such as dexamethasone. However, this kind of treatment comes along with dose-dependent side effects, which might limit the therapy efficiency in clinical. Mässenhausen et al. firstly discovered that dexamethasone sensitized ferroptosis by GSH depletion (Von Mässenhausen et al., 2022). Dialectically, it is crucial to understand the mechanisms of the side effects brought by high dose of glucocorticoids. And importantly, how can they minimize the ferroptosis damage of side effects and optimize the clinical treatment effects of dexamethasone? Meanwhile, hallmarks of COVID-19 are also associated with iron overload and problematic ROS scavenging. Due to the research we mentioned above (Meyer, 2006; Wang et al., 2021a; Kung et al., 2022), we hypothesized that ferroptosis inhibitors had potential to be a kind of adjuvant therapy, for example, could ameliorate the side effects of ferroptosis damage from the use of glucocorticoids, and impaire viral replication via iron chelation. Suggestively, multiple interactions between SARS-Cov-2 and ferroptosis may provide diverse perspectives in the exploration of strategy for COVID-19 treatment.

For the recent years, the therapeutic potential of ferroptosis was widely discussed in cancer or other diseases, excepted for infectious diseases. Although the concept of ferroptosis was officially proposed until 2012, but its relevant mechanisms had existed in many diseases for a long time. Table 1 and 2 may give us some hints or clues for the exploration of therapeutic methods. These inhibitors or inducers are involved in many basic biological processes, such as iron metabolism, redox reaction, and protein synthesis. And these processes always appear to be dysregulated in diseased conditions. And it is reasonable to seek therapy targets via three aspects: iron overload, LPO and GPX4 antioxidant system. Obviously, it is of worth to discuss the possibility of therapy for these drugs in infectious diseases, especially when close interrelationships between ferroptosis and the diseases are showed. Crucially, what can iron accumulation, lipid peroxidation damage and disrupted cellular redox contribute to the treatment of infectious diseases? The understanding of these works would further benefit the use of ferroptosis for therapies.

Additionally, a summary table of the potential therapeutic target or treatment of ferroptosis has been made up (Table 3). Notably, among these possible options discussed here are a combination of hypothesis stemming from *in vivo* or *in vitro* researches.

**TABLE 3:** Summary of the relationships between ferroptosis and infectious diseases and potential therapeutic target or treatment.

Infectious disease	Ferroptosis effects on diseases or model	Potential therapeutic treatment or target
Bacteria infection		
*Mycobacteria tuberculosis* infection	Facilitates the necrosis of infected macrophages, promotes the dissemination of Mtb *in vitro*	Inhibition of BACH-1 related ferroptosis Amaral et al. (2020), Aberman et al. (2021), the use of Fer-1 Amaral et al. (2019))
*P. aeruginosa* infection	Catalyzes lipid peroxidation in human bronchial epithelial cells, promotes the spreading to surrounding cells and tissues	Inhibition of 15-LOXes-related ferroptosis, the use of Fer-1 Ousingsawat et al. (2021)
Sepsis-induced cardiac injury	Mitochondria damage caused by ferritinophagy-related ferroptosis	Inhibition of NCOA4-related ferritinophagy, the inhibiton of ferroptosis via upregulating Nrf-2, the use of Fer-1 and Dexrazoxane (DXZ) He et al. (2022)
*S. aureus, E. coli, S. pullorum,* and *S. enteritidis* infection	Ferroptotic stress promotes macrophages against intracellular bacteria, inducing ferroptosis in bacteria (in the mouse infection models)	Enhancing ferroptotic stress with sulfasalazine suppressed bacteria Ma et al. (2022)
Listeria infection	p53-induced ferroptosis results in massive intracellular iron accumulation in macrophages, promoting infection	The inhibition of p53-induced ferroptosis Singh et al. (2020)
Viral infection		
U251 cells infected by *Newcastle-disease-virus* (NDV)	Upregulated expression of p53 leads to the decrease of SLC7A11 and GPX4 protein level; ferritinopahgy induced ferroptosis	The inhibition of p53-related ferroptosis or NCOA4-related ferritinophagy Kan et al. (2021)
*enteroviruses (CV-A6)* and *coronaviruses* (CoV-229E, CoV-NL63, CoV-OC43, CoV-HKU1, and SARS-CoV-2) infected *in vivo*	Upregulated expression of p53 leads to the decrease of SLC7A11 and GPX4 protein level; ferritinopahgy induced ferroptosis	The inhibiton of ASCL4 Kung et al. (2022)
COVID-19	Decreases the GPX4 mRNA level, upregulated hepcidin cause deranged iron metabolism, promote infection	The inhibtion of ASCL4, the use of iron chelators like DFO Meyer (2006); Wang et al. (2021a); Kung et al. (2022)
Parasite infection		
*Leishmania infection*	Infection of GPX4-deficient mice with Leishmania leads to a reduction in the number of CD4^+^ T cells, which contributes to the maintenance of Leishmania *in vivo*	Enhancement of GPX4 expression Matsushita et al. (2015)
*Malaria*	p53-induced ferroptosis leads to M2-polarization of macrophages, helping resist infection, p53-SLC7A11-GPX4 pathways resist infection in liver	Induce the mutation of amino acid 47 in human TP53, induction of p53-induced ferroptosis Singh et al. (2020)
Fungal infection		
*Cryptococcal meningitis*	Ferroptosis hallmarks were detected in clinical samples, but no studies have directly pointed out the role of ferrotptosis in cryptococcus infection	Hypothesize the inhibition of iron accumulation or lipid peroxidation may contribute to the therapy Gross et al. (2000)

## Conclusion and perspectives

For many years, soaring progresses have been made in delineating the mechanisms that modulate ferroptosis and its occurrence in different diseases (i.e., Parkinson’s, kidney diseases, cancers), however, only a visble crack can be seen in the door of infectious diseases. Therefore, bridging ferroptosis with infectious diseases is particularly on the map. As we reviewed above, multiple functions of ferroptosis in infectious diseases can be generally considered as a double-edged sword (Figure 5). On the one hand, ferroptosis poses threats to immune cells like perturbing the normal immunity performance against infections in macrophages. On the other hand, some kinds of “pro-immunity” ways of ferroptosis, such as its occurrence in pathogens inside defensing against the infection, causing the release of DAMPs as PAMPs and activating the immune responses, can facilitate the host control of infections. For example, some intracellular pathogens evade the killing of macrophages by immune escape while ferroptosis promotes macrophages to kill intracellular bacteria, indicating that ferroptosis may be used as a means of treating intracellular bacteria. However, this effect might be inversed to show impaired killing effects of host cells to the intracellular bacteria due to the dysfunction of host cell ferroptosis. Such fascinating facts undoubtedly point out the engaging connections between ferroptosis and host immunity, especially in host cells, such as macrophages. Obviously, more thorough clarifications about the modulations and functions of ferroptosis in infectious diseases are needed to be done.

Furthermore, relationships between ferroptosis-related pathways, metabolism, and host immunity against infections are also striking enough in the discovery tour. As proof, the “selenium-GPX4-ferroptosis” pathway provides a novel insight into the development and enhancement of vaccination. Meanwhile, iron homeostasis, one of the key factors of ferroptosis, to some degree is a tiger that host riding with. Excess iron-induced ferroptosis causes the damage, but on the bright side, NCOA4-mediated ferrtinophagy can possibly be utilized by macrophages to kill intracellular bacteria *via* inducing ferroptosis. Also, iron homeostasis is maintained by various proteins and transcription factors, such as iron chaperone proteins PCBP1, a negative regulator of ferroptosis. Although the connections between PCBP1 and infectious diseases haven’t been elucidated, these kinds of functional molecules may provide more inspiration for the future work in this field. Simultaneously, lipid metabolism involved in infectious diseases, such as 15-LOXes in *P. aeruginosa* catalyzed lipid peroxidation in human bronchial epithelial cells to induce ferroptosis with increased damage, is also worth to be mentioned. This means that ferroptosis-driven lipid metabolism might be a target for the development of infectious diseases. Similarly, it is reported that ROS, which induces ferroptosis, mainly comes from mitochondria (Li et al., 2021). Given the importance of mitochondrial as a multifunctional unit in managing basic physiological events and diverse host responses against infections, it makes sense to extrapolate that modulation mitochondrial functions might be a way to control ferroptosis pathways upon infections. In summary, compelling evidences indicate the complex connections among physiological and pathological metabolism, ferroptosis, and pathogen infections, which might be a new entry points of therapy in infectious diseases.

Many researchers have proposed their perspectives about the role of ferroptosis or illustrated the mechanisms involved in infectious diseases. For example, While Amaral et al. suggested the inhibition of ferroptosis might ameliorate the Mtb infection, they also considered that simultaneously lessen tissue damage while reducing pathogen burden and dissemination is an attractive aspect of this strategy (Amaral et al., 2019). And Yao et al. proposed that regulation of ferroptosis as a strategy to boost humoral immunity in infection and following vaccination (Yao Y. et al., 2021). Summarily, these diverse perspectives elicit public an open mindedness in the exploration of ferroptosis for infectious diseases treatment.

However, plenty of questions remain unknown, such as whether it is possible to develop ferroptosis as target into a new therapeutic method for infectious diseases or not. To what extent can ferroptosis assist the host killing of pathogens, and how can we minimize the damage of ferroptosis to host physiological functions? How does ferroptosis mediate the pathological progresses during the infections? How do ferroptosis-related regulations affect the innate and adaptive immunity against the infections? What are the similarities and complementarities between ferroptosis and cell death such as autophagy and necrosis in infectious diseases? Undoubtedly, tons of puzzles sitting right there need us to solve in the future. Delving into and thoroughly settling the mechanisms of ferroptosis in host upon infections is a promising, yet albeit challenging strategy to help conquer these diseases. And we believe that with the increasing understanding of the relations and underlying mechanisms between ferroptosis and infectious diseases, the regulation of ferroptosis might be developed into novel therapeutic strategy, which would further benefit the control of the threatening infectious diseases.
